# Impact of lung cancer screening on surgical stage distribution and surgical practice: a regional analysis of patients operated in and out of a screening program

**DOI:** 10.1093/icvts/ivad193

**Published:** 2023-11-24

**Authors:** Michael R Gooseman, Vasileios Tentzeris, Kerry L Bulliment, Syed S A Qadri, Matthew E J Callister, Richard Milton, Nilanjan Chaudhuri, Peter Tcherveniakov, Kostas Papagiannopoulos, Michael E Cowen, Alessandro Brunelli

**Affiliations:** Department of Cardiothoracic Surgery, Castle Hill Hospital, Hull University Teaching Hospitals, Cottingham, UK; Department of Cardiothoracic Surgery, Castle Hill Hospital, Hull University Teaching Hospitals, Cottingham, UK; Department of Cardiothoracic Surgery, Castle Hill Hospital, Hull University Teaching Hospitals, Cottingham, UK; Department of Cardiothoracic Surgery, Castle Hill Hospital, Hull University Teaching Hospitals, Cottingham, UK; Department of Respiratory Medicine, St James's University Hospital, Leeds Teaching Hospitals, Leeds, UK; School of Medicine, University of Leeds, Leeds, UK; Department of Thoracic Surgery, St. James's University Hospital, Leeds Teaching Hospitals, Leeds, UK; Department of Thoracic Surgery, St. James's University Hospital, Leeds Teaching Hospitals, Leeds, UK; Department of Thoracic Surgery, St. James's University Hospital, Leeds Teaching Hospitals, Leeds, UK; Department of Thoracic Surgery, St. James's University Hospital, Leeds Teaching Hospitals, Leeds, UK; Department of Cardiothoracic Surgery, Castle Hill Hospital, Hull University Teaching Hospitals, Cottingham, UK; School of Medicine, University of Leeds, Leeds, UK; Department of Thoracic Surgery, St. James's University Hospital, Leeds Teaching Hospitals, Leeds, UK

**Keywords:** Lung cancer screening, Non-small-cell lung cancer, Surgery, Segmentectomy

## Abstract

**OBJECTIVES:**

The aim of this study was to assess variations in surgical stage distribution in 2 centres within the same UK region. One centre was covered by an active screening program started in November 2018 and the other was not covered by screening.

**METHODS:**

Retrospective analysis of 1895 patients undergoing lung resections (2018–2022) in 2 centres. Temporal distribution was tested using Chi-squared for trends. A lowess curve was used to plot the proportion of stage 1A patients amongst those operated over the years.

**RESULTS:**

The surgical populations in the 2 centres were similar. In the screening unit (SU), we observed a 18% increase in the proportion of patients with clinical stage IA in the recent phase compared to the early phase (59% vs 50%, *P* = 0.004), whilst this increase was not seen in the unit without screening. This difference was attributable to an increase of cT1aN0 patients in the SU (16% vs 11%, *P* = 0.035) which was not observed in the other unit (10% vs 8.2%, *P* = 0.41). In the SU, there was also a three-fold increase in the proportion of sublobar resections performed in the recent phase compared to the early one (35% vs 12%, *P* < 0.001). This finding was not evident in the unit without screening.

**CONCLUSIONS:**

Lung cancer screening is associated with a higher proportion of lung cancers being detected at an earlier stage with a consequent increased practice of sublobar resections.

**Figure:**
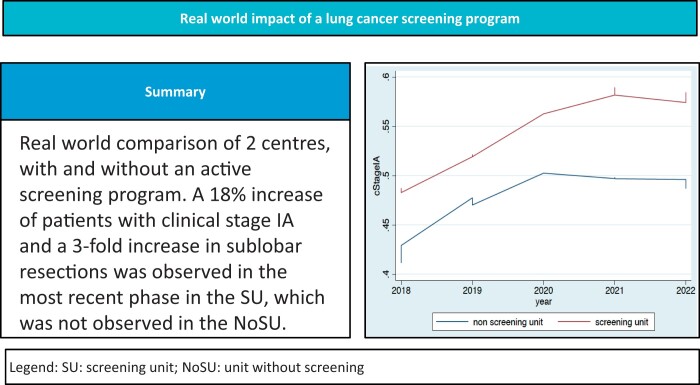

## INTRODUCTION

Lung cancer continues to be a major cause of mortality with a 5-year survival rate just in excess of 20% [1]. This is on the background of intensive activity over recent years to improve outcomes from this disease. The progress reflects advancement in precision therapy for lung cancer such as immunotherapy, ongoing refinement in surgical technique and the continued implementation of lung cancer screening programs.

The National Lung Screening Trial and the Dutch-Belgian Randomized Lung Cancer Screening Trial (NELSON) study demonstrated how CT screening effectively improved survival from lung cancer with much of the reduction in mortality as a result of increased detection of stage 1 disease [2, 3] After patients are identified with stage 1 disease there is not a single treatment modality and there are, of course, numerous factors that contribute to the decisions made in this respect. In those patients proceeding to surgical resection, the previously considered gold standard of pulmonary lobectomy may no longer be seen as such.

The role of pulmonary segmentectomy has come under renewed scrutiny as a result of smaller lesions being detected through screening. The JCOG-0802 randomized trial demonstrated that anatomic segmentectomy is associated with similar, if not, superior outcomes when compared with lobectomy for solid or part solid early-stage lung cancer [4]. We wanted to investigate what the impact of a local screening program had in terms of the surgical stage and the impact on surgical practice.

## PATIENTS AND METHODS

### Ethical statement

The study was reviewed by the Institutional Review Board of the Leeds Teaching Hospitals, which classified the study as service evaluation not requiring formal NHS Research Ethics Committee review and waived patients’ consent as retrospective and anonymous data were analysed.

This is retrospective multicentre analysis performed on data extracted from prospectively maintained institutional databases normally used for clinical service evaluation. All patients who underwent lung resection for non-small-cell lung cancer (NSCLC) in 2 different NHS Trust hospitals (St. James’s Hospital, Leeds, and Castle Hill Hospital in Hull) from January 2018 through December 2022 were analysed.

The 2 hospitals are located in the same UK County, Yorkshire and Humber. The 2 thoracic surgery services are the only centre performing thoracic surgery in those 2 different regional catchment areas and have a similar surgical volume. The Leeds thoracic surgery unit serves a total population of 2.7 million in the West Yorkshire, and the Hull thoracic surgery unit serves a total population of 1.2 million in the East Yorkshire.

Thoracic services are centralized in the UK and each hospital receives referral from their specific referral area.

According to national regulations in UK all patients referred for suspected or proven lung cancer are discussed in multi-disciplinary meetings lead by the respiratory physicians and involving multiple specialists (respiratory physicians, medical oncologists, pathologists, radiologists, thoracic surgeons, radiation oncologists, lung cancer nurses).

Diagnostic and staging pathways and policies are similar in the 2 centres as mandated by national guidelines [5, 6]. All patients with suspected lung cancer are staged with a positron emission tomography (PET)–computed tomography (CT) and tissue diagnosis is attempted preoperatively in all cases whenever deemed feasible and using the most appropriate approach (CT-guided biopsy, navigational bronchoscopy biopsy, endo bronchial ultrasound (EBUS), esophageal ultrasound (EUS). About 50% of patients had a preoperative tissue diagnosis of lung cancer. Ninety-five percent of patients were confirmed to have lung cancer after surgery.

The most appropriate curative treatment is discussed and agreed in the multi-disciplinary team (MDT) meeting. For borderline patients with increased surgical risk due to fitness limitations, a parallel referral to both the surgeon and radiation oncologist is often organized. The proportion of stage I patients who were not treated surgically among the screening patients was 30%.

The thoracic surgeons working in the 2 units attend multiple local MDTs in their own respective areas, but surgery is performed only in St James’s or in Castle Hill Hospitals, respectively. Patients are referred to the Lung cancer teams through different routes. In both centres, most of the patients are referred from the primary care services, or directly from the Emergency Departments in case of severe symptoms. In Leeds, a large randomized lung cancer screening trial, Yorkshire Lung Screening Trial, was funded by the Yorkshire Cancer Research and commenced in November 2018 and completed the second round of biennial screening in December 2022 [7]. The study only paused for 3 months in 2020 due coronavirus disease 2019 pandemic. More than 45 000 ever-smoked people and aged between 55 and 80 years were invited if they met one of the following eligibility criteria: the prostate, lung, colorectal and ovarian (PLCO) risk ≥1.51% [8], liverpool lung project (LLP) risk ≥5% [9] or the U.S. preventive services task force (USPSTF) criteria [10]. The response rate was 51% with >7000 people screened.

Although the UK does not yet have a comprehensive rolling lung cancer screening programme, additional targeted local lung health checks are offered in some part of the country to ever-smoked people between 55 and 74 years of age and registered with a primary care practice.

This type of service has started to be offered in the Bradford District, which is within the catchment area of the Leeds thoracic service since the end of 2022.

A similar service was commenced in Hull early 2020 but then stopped for the coronavirus disease outbreak. It only fully resumed beginning of 2022. Only about 1.6% of the patients operated in Hull were referred through a local lung health check program.

In both centres, patients referred for surgery from the MDTs met and were operated by qualified thoracic surgeons who were in charge to choose the most appropriate approach and extent of resection.

For the purpose of this study, the Leeds unit was defined as the screening unit (SU) and the Hull unit was the non-screening one (NoSU).

### Statistical analysis

A comparison of baseline and surgical variables between centres was performed using standardized differences for both continuous and categorical variables.

Clinical staging was based on the 8th edition of the tumour-node-metastasis staging system. For the purpose of the present analysis, the study period was divided into an early phase (2018–2019), when screening was just started in Leeds and a late phase (2021–2022) when the screening program was well established and its effect on the surgical case-mix would be evident.

Variation in stage distribution and in the rate of sublobar resections within each unit between different time phases was assessed using Chi-squared.

Lowess curves were used to plot the proportion of stage 1A patients among all operated patients over the years in each centre and to plot the incidence of sublobar resection among all types of resections over the years in each centre.

In order to account for other confounders, a logistic regression was performed in which the dependent variable was the number of sublobar resection and independent ones were age, gender, forced expiratory volume in 1 s, body mass index, the year of operation, being in clinical stage IA and the being the unit associated with screening.

All tests were performed using the Stata 15.0 statistical software (Stata Corp, College Station, TX, USA).

## RESULTS

A total of 1895 patients were operated in the 2 centres for NSCLC between January 2018 and October 2022. One thousand and ninety-five patients were operated in the screening centre whilst 800 were operated in the centre without screening. About 13% of patients operated in the SU were referred from the screening program during the period of the study.

Table 1 shows the characteristics of the patients included in this study. Patients operated in the 2 centres were substantially similar as shown by the standardized differences <0.2 with the exception of a higher rate of minimally invasive surgery in the SU.

**Table 1:** Characteristics of the patients included in the study

Variables	Total	Screening unit (*n* = 1095)	Non-screening unit (*n* = 800)	Standardized difference	*P*-value
Age	69.3 (8.3)	69.8 (8.2)	68.7 (8.6)	0.13	0.004
Gender male, *n* (%)	865	499 (46%)	366 (46%)	0.004	0.93
Ever smoker, *n* (%)	1697 (90%)	997 (91%)	700 (88%)	0.19	0.015
BMI (kg/m^2^)	27.2 (5.2)	27.2 (5.1)	27.2 (5.3)	0.008	0.73
FEV1%	89.7 (20.5)	90.7 (21.0)	88.1 (19.6)	0.13	0.024
CAD	229	112 (10%)	117 (15%)	0.13	0.004
CVD	105	39 (3.6%)	66 (8.3%)	0.199	<0.001
MIS, *n* (%)	1493	926 (85%)	567 (71%)	0.33	<0.001
Pneumonectomies, *n* (%)	67	46 (4.2%)	21 (2.6%)	0.09	0.067

Results are expressed as means and standard deviations for numeric variables and as count and percentages for categoric variables.

BMI: body mass index; CAD: coronary artery disease; CVD: cerebrovascular disease; FEV1: forced expiratory volume in 1 s; MIS: minimally invasive surgery.

Seven hundred and eighty-six patients were operated in the early phase (2018–2019), 328 in the NoSU and 458 in the SU. A total of 801 patients were instead operated in the most recent phase (2021–2022), 363 in the NoSU and 438 in the SU.

In the NoSU, a similar proportion of patients in the early and in the recent phase were in clinical stage IA at the time of surgery (47% vs 50%, *P* = 0.32).

In the SU, an 18% increase in the proportion of patients with clinical stage IA was observed in the recent phase compared to the early phase (59% vs 50%, *P* = 0.004).

**Figure 1:**
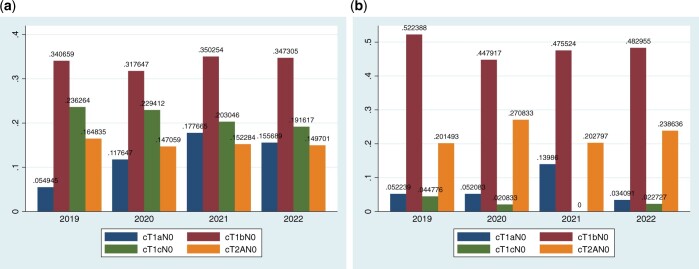
Stage distribution of clinical stage IA NSCLC patients in the unit with (A) and without (B) screening across the years. NSCLC: non-small-cell lung cancer.

Figure 1a and b shows the stage distribution of clinical stage IA patients in the 2 units across the years.

Most of the changes observed in the recent phase in the SU are attributable to an increase in stage T1aN0 (16% vs 11%, *P* = 0.035) which is not observed in the NoSU (10% vs 8.2%, *P* = 0.41). In the SU, the proportion of patients with clinical stage IA1 increased by 46% from the early to the recent phase.

**Figure 2:**
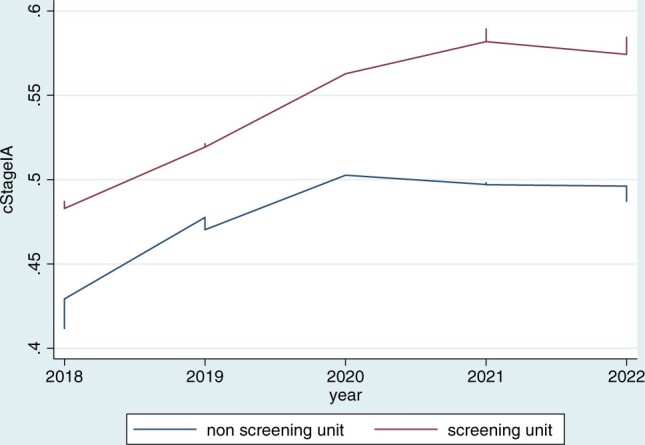
Lowess plots showing the proportion of surgical patients with clinical stage I in the 2 centres over the years.

Figure 2 plots the proportion of surgical patients with clinical stage I in the 2 centres over the years. It is evident that, in the SU, there is a more consistent and progressive increase of this proportion over the years compared to the NoSU.

Table 2 shows the general characteristics, tumour stage and type of resection in the 2 units during the different periods.

**Table 2:** General and surgical characteristics of patients in the 2 units operated in the 2 periods

	Screening unit		Unit without screening	
	Early period (*n* = 458)	Late period (*n* = 438)	Early period (*n* = 328)	Late period (*n* = 363)
Age	70.1 (8.4)	69.8 (8.0)	68.3 (8.7)	69 (8.3)
FEV1%	90.2 (21.4)	90.2 (21.3)	87.6 (20.5)	89.2 (19.1)
Gender male, *n* (%)	221 (48%)	189 (43%)	155 (47%)	153 (42%)
Clinical stage IA	228 (50%)	260 (59%)	153 (47%)	183 (50%)
Sublobar resections, *n* (%)	55 (12%)	155 (35%)	92 (28%)	115 (32%)

Results are expressed as means and standard deviations for numeric variables and count and percentage of total for categoric variables.

FEV1: forced expiratory volume in 1 s.

Consistent with the above findings, in the SU, there was also a three-fold increase in the proportion of sublobar resections performed in the recent phase compared to the early one (35% vs 12%, *P* < 0.001). We were not able to detect this increase in the NoSU, where the rate of sublobar resection remained stable between the early and recent phase (28% vs 32%, *P* = 030). Nevertheless, if the analysis was limited to clinical stage IA patients only, we still observed an increased rate of sublobar resections in the SU in the late period compared to the early one (50% vs 26%, respectively, *P* < 0.001). The same was not observed in the NoSU in which the rate remained stable within clinical stage IA patients between the 2 periods (43% vs 41%, *P* = 0.73).

**Figure 3:**
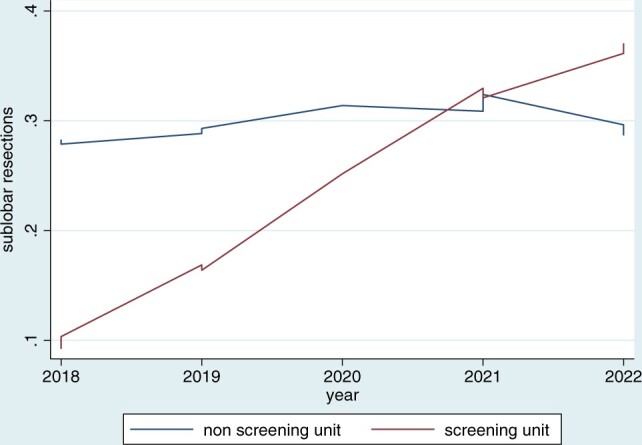
Lowess plots showing the rate of sublobar resections in the 2 centres over the years.

Figure 3 plots the rate of sublobar resections in the 2 centres over the years, showing a consistent increase in the SU.

After adjusting for other confounders with a logistic regression analysis, being in clinical stage IA [odds ratio (OR) 6.0, 95% confidence interval (CI) 4.1–8.6, *P* < 0.001] and the year of surgery (OR 1.5, 95% CI 1.4–1.7, *P* < 0.001) remained independently associated with increased rate of sublobar resections in the SU. Only being in clinical stage IA (OR 3.1, 95% CI 2.2–4.5, *P* < 0.001) but not the effect of time (OR 1.03, 95% CI 0.91–1.16, *P* = 0.63) was associated with the number of sublobar resections performed in the unit without screening.

Similar to clinical stage, in the SU, there was a 21% increase of patients with pathologic stage IA in the recent phase compared to the early one (41% vs 34%, *P* = 0.035). The rate of patients with stage IA at definitive pathologic examination remained stable in the NoSU (49% vs 51%, *P* = 0.62).

## DISCUSSION

There is now a strong body of evidence to support the use of lung cancer screening in improving lung cancer survival. This is in large part as a result of the increased detection of stage 1 disease which is consistent with the findings of our study with an almost 10% increase in the T1a lesions in the SU. Large prospective trials including the National Lung Screening Trial and NELSON have shown that the reduction in lung cancer was mainly as a result of the detection of stage 1 disease. For instance, in the NELSON trial 47% of NSCLC diagnosed in the male population of the screening group were in stage IA compared to less than 7% in the control group [3]. This proportion was remarkably similar to the one observed in the SU in our study where 59% of patients were in stage IA. The higher proportion in our study may be explained by the fact we only included surgical patients. In addition, the UKLS trial also found a proportion of stage IA patients of about 53% in the screened population compared to 14.5% in the control group [11]. Other trials had produced various results, but a meta-analysis was recently performed of all published randomized clinical trials (RCTs) incorporating over 90 000 participants and a 16% relative reduction in lung cancer mortality was established [11].

Although our study did not assess the relative impact of screening on long-term outcomes the increase of proportion of early-stage diagnosis would likely translate into improved survival.

With the increased implementation and adoption of lung screening and earlier detection of disease, there has been a lot of debate regarding the form of surgical resection. Whilst surgery is recognized as the gold standard for early-stage disease, there has recently been intense debate and interest as to what the optimal resection approach should be.

Pulmonary lobectomy has for the last few decades been the operation of choice for early-stage lung cancer and supported by guidelines as the recommended resection; this itself ultimately a reflection of the Lung Cancer Study Group that is now nearly 30 years out following publication [12]. This study demonstrated superiority of lobectomy over sublobar resection in terms of recurrence rate. However, the JCOG-0802 randomized trial has confirmed segmentectomy is associated with at least similar or even superior overall survival when compared to lobectomy for early-stage lung cancer (<2 cm) [4] This led to a recommendation from the authors that segmentectomy should become the standard resection for early-stage lung cancer.

The plan for the health service in the UK is to increase the detection of all cancers at an early stage by 25% over the next 5 years [13] Therefore, it is reasonable and logical to assume that the detection of T1a and smaller lung cancers will continue to increase with screening as this study has also demonstrated. Pulmonary segmentectomy is therefore going to continue becoming an increasingly important operation that will need to be undertaken at increasing rates by all thoracic surgeons.

Our study has shown that there was a three-fold increase in the rate of pulmonary segmentectomy performed in the SU. The decision to proceed to operation is from the MDT after full discussion with and agreement from the patient. However, the type of resection is largely based on surgical judgement incorporating various factors. Surgical experience is likely to be a key element of this. Segmentectomy has often been felt to be a more challenging operation than lobectomy with higher rates of technical complications such as air leak. With the proven benefit of minimally invasive lung resection, thoracoscopic segmentectomy has different challenges compared to lobectomy. These include factors such as the dissecting areas being more in the periphery as opposed to in the hilum. Furthermore, identifying and then dividing the intersegmental plane is of course unique to segmentectomy—understanding how to position and control mechanical staplers is something that needs to be considered. Also, the approach to lymph node dissection in segmentectomy needs to be learnt and understand. Therefore, the point of ensuring adequate training in thoracic surgeons to undertake all forms of segmentectomy arises. There is increasing emphasis on training in segmentectomy using things such as 3-dimensional modelling but training programs may need to be more formalized as guidance updates potentially recommending the role of segmentectomy. In the current study, the increase in sublobar resections in the SU in the most recent period was 3-fold compared to the early phase. This is likely the combined effect of an increased adoption of this procedure by the surgical team (given the fact we observed a doubling of sublobar resections in clinical stage IA patients in the late period) along with an increased rate of early-stage lung cancer detected through screening. This explanation is further supported by the fact that being diagnosed with a clinical stage IA NSCLC was independently associated with increased number of sublobar resections in both centres, whereas most recent years of the operation were significantly associated with the number of sublobar resections only in the unit with screening.

### Limitations

This study may have some limitations. First, this is a retrospective study with inherent selection bias. As mentioned in the discussion, surgical experience is likely to have played a role in the findings noted—the majority of segmentectomies performed in the SU were by a single surgeon. It is also on the backdrop of the current national standard of care during this timeframe that there is no recommendation for undertaking segmentectomy. Furthermore, this study incorporated only 2 centres being directly compared and other unaccounted factors may have played a role in the stage and surgical variation during the study period. For example, other uncontrolled factors may have influenced the increased adoption of segmentectomies in the SU, such as a deliberate change in surgical protocol, facilitated by an upsurge of early-stage lung cancers referred to surgery from screening.

In addition, the incidence of sublobar resection in the unit without screening was already particularly high in the early phase which may have limited a further increase of this rate.

It is worth to mention that in the SU only half of the eligible population were screened over the study period, with the other half in the control group. This means that the effect on staging and surgical activity might be half what might be expected with a fully rolled-out screening programme.

Our study focussed on the impact of lung cancer screening on stage and surgical procedure distribution. Other important aspects inherent to a lung cancer screening program were not assessed. For instance, data were not available at the time of the study to determine the cumulative impact of screening on the total volume of lung cancer resection performed in the 2 centres over the year. Similarly, the financial implications and resource allocation determined by the screening program will need a specific evaluation to determine its cost-effectiveness in individual hospital settings. It is interesting to note that the overall surgical volume remained substantially stable in the SU whilst increased marginally in the NoSU. Important structural and capacity factors may have certainly played a role to explain this apparent discrepancy. Further analysis will be critical to produce figures to be used for negotiating adequate resource allocation to cope with the anticipated increase in surgical demand (i.e. need for more ward beds, staff, theatre spaces, outpatient clinics slots, etc.). Finally, future analyses with a longer time frame are needed to assess the impact of this program on cancer-specific survival in different regional areas.

## TAKE HOME MESSAGE

This study demonstrates that in a real-world analysis of patients operated in a screening programme, there is a significant shift to an earlier stage. A consequence of this, and of importance to the thoracic surgeon, an increased rate of pulmonary segmentectomy is observed. It is therefore important that thoracic surgeons ensure they are equipped and confident to regularly undertake this operation.

## Data Availability

The data underlying this article will be shared on reasonable request to the corresponding author.

## Notes

Presented at the 31st European Conference on General Thoracic Surgery, Milan, Italy, 4–6 June 2023.

## ABBREVIATIONS

CI: Confidence interval
MDT: Multi-disciplinary team
NSCLC: Non-small-cell lung cancer
OR: Odds ratio
SU: Screening unit
